# Decreased circulating and neutrophil mediated VEGF-A_165_ release in stable long-term cardiac transplant recipients

**DOI:** 10.1186/s13221-015-0029-8

**Published:** 2015-04-22

**Authors:** Damien Vitiello, Diana Chaar, Paul-Eduard Neagoe, Anique Ducharme, Michel Carrier, Guy B Pelletier, Normand Racine, Mark Liszkowski, Martin G Sirois, Michel White

**Affiliations:** Research Center, Montreal Heart Institute, Montréal, Qc Canada; Université de Montréal, 5000 Belanger Street, Montreal, QC Canada

**Keywords:** Allograft, Cardiac transplantation, Inflammation, Interleukin-1 recipient antagonist, Neutrophils, VEGF-A_165_

## Abstract

**Background:**

Vascular endothelial growth factor (VEGF) may play a role on the allograft remodelling following cardiac transplantation (CTx). We measured the circulating levels of VEGF-A_165_ concomitantly with the proinflammatory (Interleukin-8; IL-8), anti-inflammatory (IL-1 receptor antagonist; IL-1RA) and their release from neutrophils of CTx recipients.

**Methods:**

Eighteen CTx recipients aged 49.6 ± 3.1 years, being transplanted for 145 ± 20 months were age-matched to 35 healthy control (HC) subjects. Concomitantly to plasma assessment, circulating neutrophils were isolated, purified and stimulated by vehicle (PBS), N-formyl-Met-Leu-Phe (fMLP, 10^−7^ M), bacterial lipopolysaccharide (LPS, 1 μg/mL), or tumour necrosis factor alpha (TNF-α, 10 ng/mL).

**Results:**

Compared with HC, CTx recipients exhibited a decrease (−80%) in plasmatic levels of VEGF-A_165_ (225 ± 42 (HC) vs 44 ± 10 pg/mL (CTx); (p < 0.001). There were no differences in the levels of IL-8 and IL-1RA. Under basal or stimulated conditions, neutrophils from CTx patients exhibited a marked decrease ranging from −30 to −88% on their capacity to release VEGF-A_165_, IL-8 and IL-1RA upon stimulation.

**Conclusions:**

Long-term CTx recipients exhibit a marked reduction in the circulating levels of VEGF-A_165_, as well as neutrophil-mediated release of VEGF-A_165_, IL-1RA and IL-8 compared to healthy volunteers. The mechanisms and physiological impacts of these findings deserve additional investigations.

## Background

De novo [1] and long-term cardiac [2] transplantation (CTx) are characterised by an increase in the circulating markers of subclinical inflammation and oxidative stress. Selected biomarkers related to these processes remain elevated more than 8 years following cardiac transplantation [2]. Coronary allograft vasculopathy (CAV) exhibits distinct histologic and pathophysiologic features [3]. Also a chronic state of inflammation likely contributes to CAV but also to the long-term vascular complications following solid organ and cardiac transplantation [4,5].

Although multiple growth factors induce the proliferation and migration of endothelial cells (EC), vascular endothelial growth factor (VEGF) is the only growth factor capable to promote protein extravasation which is linked to its angiogenic properties [6-8]. There are five different VEGF-A isoforms, of various members of amino acids, termed as VEGF-A_206, 189, 165, 145 and 121_, which are produced from a single gene by alternative splicing. The VEGF family also includes five different analogs: placental growth factor (PlGF-1 and −2), VEGF-B, VEGF-C, VEGF-D, and a viral homolog, VEGF-E [6-9]. In addition, neuropilin-1 (NRP-1), a transmembrane receptor, acts as a coreceptor, complexing with VEGF receptors (VEGFR-1 and VEGFR-2) [10-13]. NRP-1 specifically enhances the binding of VEGF-A_165_ to VEGFR-2 and potentiates various VEGF-A_165_ biological activities [11]. The most efficient VEGF analog capable to promote biological activities is mainly driven by VEGF-A_165_ isoform. Chronic cardiac hypoxia likely contributes to molecular remodeling in the transplanted human hearts [14-16]. In fact, cardiac VEGF-A isoforms increase concomitantly with the presence of fibrin depositions [17] and endomyocardial fibrosis following CTx [16]. Also more recent investigations reported an increase in circulating VEGF-A_165_ and VEGF-C in patients with CAV. However data were not compared with healthy control subjects [18].

The neutrophil plays a significant role on vascular proinflammatory responses [19,20]. Data generated from model systems showed that the neutrophil mediated smooth muscle cell loss precedes CAV [21]. Neutrophils can promote the release of various interleukins such as IL-1, IL-6 and IL-8, which may play a significant role on the vascular inflammatory microenvironment and consequently on the long-term complications following organ transplantation [22-24]. Although VEGF-A mRNA and protein isoforms have been measured in cardiac tissue following CTx [16,17], the assessment of circulating levels of VEGF-A_165_, but also levels of pro- and anti-inflammatory cytokines concomitantly with the evaluation of neutrophil mediated inflammatory response have not been investigated in stable long-term cardiac transplant recipients.

The primary objective of this investigation was to assess the circulating levels of VEGF-A_165_, IL-8 and IL-1RA concomitantly with the study of basal and stimulated neutrophil’s proinflammatory response in stable long-term CTx recipients. The secondary objective of this study was to explore the impact of coronary allograft vasculopathy (CAV) on these responses.

## Methods

### Study population

Eighteen CTx recipients and 35 age-matched healthy control (HC) subjects were recruited. Patients were eligible if they were clinically stable and received stable doses of immunosuppressive drugs for at least 4 weeks prior to enrolment in the study. The most significant exclusion criteria included recent cardiac rejection, active infection and any clinically significant inflammatory condition such as arthritis or recent surgery. In addition, patients with significant anemia (hemoglobin ≤90 g/L) poorly controlled diabetes mellitus (glycated hemoglobin ≥ 10%) or systemic hypertension, active cancer (other than skin cancer), and severe renal failure (creatinine clearance less than 15 ml/min/m^2^) were excluded. Patients were recruited regardless of the presence or absence of cardiac allograft vasculopathy (CAV). Patients had to present stable or no symptoms for at least 3 months, and CAV was diagnosed by coronary angiography. The HC group had to be free from any medical condition or medication for at least 10 days prior to the experiments. The study was conducted in accordance with the Declaration of Helsinki and approved by the Montreal Heart Institute’s ethical committee (Montreal, QC, Canada; ethics No. ICM #01-406 and No. ICM #12-1374). All HC and CTx provided written informed consent to the experimental protocol before participating in the study.

### Plasma biomarkers

Venous blood samples from both HC and CTx patients were collected in the morning using the anticoagulant Citrate Dextrose Solution USP (ACD) Formula A. Two milliliters of plasma were centrifuged (11000 g, 2 min, 4°C) to obtain platelet-free plasma [25] and the samples were immediately frozen at −80°C. Plasma levels of VEGF-A_165_ (with no-cross reactivity for other native VEGF-A isoforms or VEGF analogs), IL-1RA and IL-8 were further analyzed by ELISA using the R&D Systems Quantikine kits (DVE00, DRA00B and HS800 respectively; Minneapolis, MN, USA).

### Neutrophil isolation and purification

One hundred mL of venous blood was drawn in accordance with the guidelines of the Montreal Heart Institute’s ethical committee. Neutrophils were isolated by using Ficoll-Hypaque gradient and re-suspended in RPMI medium (Lonza, Basel, Switzerland) supplemented with 25 mM Hepes (N-2-hydroxyethylpiperazine-N’-2-ethanesulfonic acid) and 1% penicillin/streptomycin as previously described [26,27]. Contamination of isolated neutrophil suspension with peripheral blood mononuclear cells was less than 0.1% as determined by morphological analysis and flow cytometry, and viability was found to be >98%, as assessed by Trypan blue dye exclusion assay.

### Neutrophil stimulation and treatments

Purified neutrophils (5×10^6^/mL, 500 μL) were incubated in RPMI-1640 solution (Gibco, Carlsbad, CA) supplemented with 5% fetal bovine serum (PAA Laboratories, Etobicoke, ON), 1% penicillin/streptomycin/GlutaMAX (P/S) (Gibco) and 25 mM HEPES (Sigma, Oakville, ON), and termed RPMI (for complete RPMI-1640 solution). Neutrophils were then stimulated for 2 and 24 hours with control vehicle (PBS), N-Formyl-Met-Leu-Phe (fMLP; 10^-7^ M) (Sigma, Oakville, ON), bacterial lipopolysaccharide (LPS; Escherichia coli 0111:B4; 1 μg/mL) (Sigma, Oakville, ON) or tumor necrosis factor-α (TNF-α; 10 ng/mL) (Peprotech, Rocky Hill, NJ) at 37°C, 5% CO_2_. Upon neutrophil stimulation, cells were centrifuged at 900 g for 7 minutes and supernatants stored at −80°C for future quantifications by ELISA of VEGF-A_165_, IL-1RA and IL-8 (R&D Systems). The selected aforementioned agonists (*i.e.* fMLP, LPS or TNF-α) were used because of their capacity to promote VEGF-A_165_, IL-1RA and IL-8 release by the neutrophils [28-31].

### Statistical analyses

All results were expressed as mean ± SEM statistical comparisons were made by a one-way analysis of variance (ANOVA), followed by a Bonferroni *t*-test. Software used was StatView 5.0 and GraphPad Prism5.0. Differences were considered significant at p values ≤ 0.05.

## Results

The clinical characteristics of the study population are presented in Table 1. A total of 53 subjects were enrolled including 18 cardiac transplant recipients (CTx) and 35 age-matched healthy controls (HC). All CTx were male and the CTx patients were studied 145 ± 20 months following transplantation. More than 60% of our transplanted studied patients exhibited treated hypertension (n = 12) and dyslipidemia (n = 11) and 39% of them (n = 7) exhibited CAV. Mean creatinine clearance measured by the MDRD formula was 67.4 ± 19.7 ml/min/m^2^ (median 66.8; 25.5 – 107). Angiography was used in all patients to assess for CAV. All patients with CAV exhibited CAV1. By design, all the patients received stable immunosuppressive drug doses for at least 4 weeks before their enrolment in the study. The majority of patients received the combination of tacrolimus (TAC) and mycophenolate mofetil/enteric-coated mycophenolic acid (MMF/EC-MPA).

**Table 1:** **Clinical characteristics of the study population**

**Clinical variables**	**CTx recipients (** ***n*** **= 18)**	**Healthy controls (** ***n*** **= 35)**
Age (years)	49.6 ± 3.1	49.3 ± 1.6
Male	18 (100%)	16 (46%)
Body mass index (kg/m^2^)	26.5 ± 0.8	
Donor age (years)	24.4 ± 3.2	
Time since transplantation (months)	145 ± 20	
***Primary diagnosis; n (%)***		
CAD	7 (39%)	
Cardiomyopathy	9 (50%)	
Other	2 (11%)	
***Medical conditions post-transplant; n (%)***		
Hypertension	12 (67%)	
Diabetes mellitus	5 (28%)	
Dyslipidemia	11 (61%)	
CAV	7 (39%)	
***Medications; n (%)***		
Statins	17 (94%)	
ACEi	5 (28%)	
ARBs	7 (39%)	
β-blockers	6 (33%)	
Calcium channel blockers	14 (78%)	
***Immunosuppressive treatments; n (%)***		
Cyclosporine A	1 (0.06%)	
Tacrolimus	15 (83%)	
Sirolimus	3 (17%)	
MMF/MPA	13 (72%)	
Prednisone		
Yes (%)	4 (22%)	
No (%)	14 (78%)	

ACEi: Angiotensin-converting enzyme inhibitor; ARBs: angiotensin II receptor blockers; CAD: Coronary artery disease, CAV: Cardiac allograft vasculopathy, MMF: Mycophenolate mofetil; MPA: Mycophenolate acid; NYHA: New York heart association. variables are expressed as mean ± SEM and percentages.

### Biomarkers

**Figure 1:**
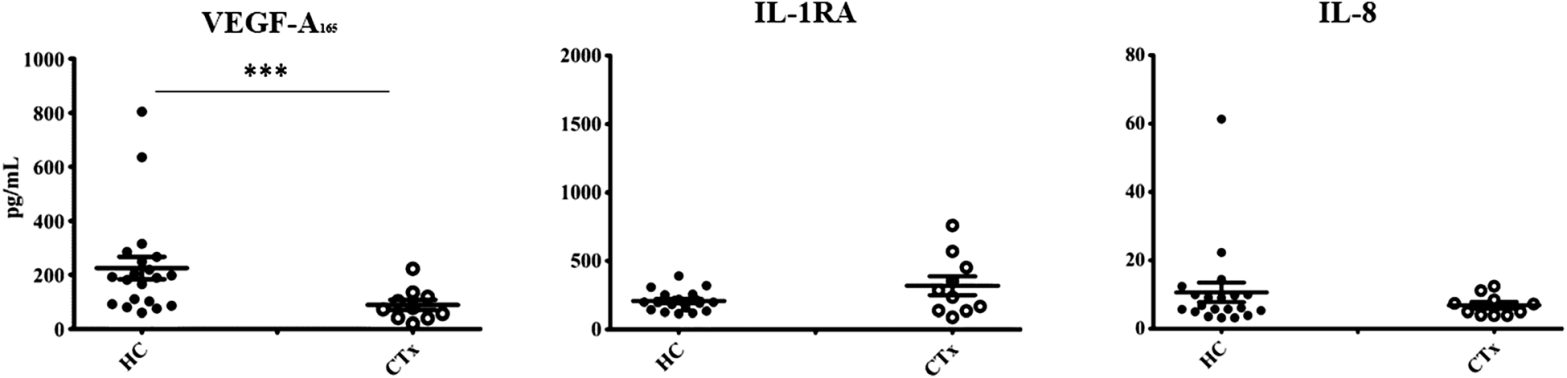
Plasma level of vascular endothelial growth factor (VEGF-A_165_), Interleukin-1 receptor antagonist (IL-1RA), and Interleukin-8 (IL-8). Data are presented as mean ± SEM. ***p < 0.001 as compared to plasma level between healthy controls (HC) and cardiac transplant recipient (CTx).

Plasma levels of VEGF-A_165_, IL-1RA and IL-8 are presented in Figure 1. Compared with the healthy control subjects, CTx recipients exhibited an 80% decrease in circulating levels of VEGF-A_165_ (225 ± 42 (HC) vs 44 ± 10 pg/mL (CTx); p < 0.001). In contrast there were no significant differences in the circulating levels of the anti-inflammatory cytokine IL-1RA (205 ± 16 (HC) vs 243 ± 45 pg/mL (CTx)) and the proinflammatory cytokine IL-8 (7.90 ± 1.05 (HC) vs 4.62 ± 0.80 pg/mL (CTx)) between both groups. There were no significant differences between patients with or without CAV (Table 2).

**Table 2:** **Circulating biomarkers and neutrophils stimulation profiles for patients with and without coronary allograph vasculopathy (CAV)**

**Parameters**	**CAV (** ***n*** **= 7)**	**No CAV (** ***n*** **= 11)**
***Biomarkers***		
VEGF-A_165_	63.3 ± 27.8	48.4 ± 15.1
IL-8	6.20 ± 1.59	3.61 ± 0.74
IL-RA	331 ± 99	187 ± 30
***Neutrophils/Factors***		
VEGF release		
PBS	10.5 ± 2.8	11.7 ± 1.9
fMLP	47.1 ± 9.5	52.7 ± 9.3
LPS	33.4 ± 5.5	46.7 ± 8.9
TNF-α	51.7 ± 11.4	60.7 ± 10.6
IL-8 release		
PBS	23.7 ± 5.3	14.7 ± 2.8
fMLP	115 ± 19	161 ± 20
LPS	1100 ± 115	1180 ± 67
TNF-α	728 ± 81	898 ± 76
IL-1RA release		
PBS	187 ± 43	229 ± 44
fMLP	178 ± 43	248 ± 42
LPS	253 ± 52	357 ± 54
TNF-α	457 ± 66	515 ± 55

CAV: Cardiac allograft vasculopathy; VEGF: Vascular endothelial growth factor; IL: Interleukin; RA: Receptor antagonist; PBS: Vehicule; fMLP: N-formyl-Met-Leu-Phe; LPS: Bacterial lipopolysaccharides; TNF: Tumor necrosis factor. Variables are expressed as mean ± SEM and percentages.

### Neutrophil responses

**Figure 2:**
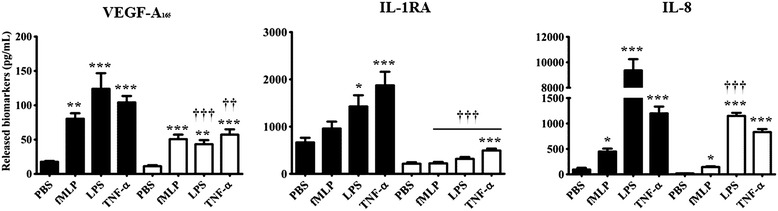
Neutrophil mediated release of VEGF-A_165_, IL-1RA, and IL-8 in response to various agonists.  healthy controls;  cardiac transplant recipients (CTx). fMLP (10^−7^ M); LPS (1 μg/mL); TNF-α (10 ng/mL). Data are presented as mean ± SEM. *p < 0.05; ***p < 0.001 compared to PBS-treated neutrophils. †††p < 0.001 vs healthy controls.

There was a marked reduction in the capacity of neutrophils from CTx patients to promote the release of all 3 cytokines either under PBS basal condition or upon stimulation with proinflammatory mediators (fMLP; 10^−7^ M, LPS; 1 μg/mL and TNF-α; 10 ng/mL) (Figure 2). The quantification of VEGF-A_165_ and IL-1RA proteins was performed after 2 hours post-stimulation with the aforementioned proinflammatory agonists, whereas, the quantification of IL-8 protein was assessed after 24 hours of treatment. All 3 agonists (fMLP, LPS and TNF-α) were nearly equivalent to promote VEGF-A_165_ release compared with PBS-treated neutrophils, ranging from 4.6 to 7.0-fold increase in HC. In neutrophils from CTx patients, we observed a similar pattern in the capacity of the agonists to increase VEGF-A_165_ release (increase ranging from 3.8 to 5.0-fold) as compared to PBS-treated neutrophils. Nevertheless, the basal level of VEGF released by the neutrophils from CTx patients was reduced by 36% as compared to HC neutrophils. In HC, IL-1RA increased by 2.2- to 2.8-fold in response to LPS and TNF-α respectively as compared to PBS*.* In contrast CTx patients yielded a significantly lesser increase in IL-1RA (1.5- and 2.3-fold by LPS and TNF-α respectively).

The release of IL-8 was increased in response to 3 agonists in HC. In contrast CTx patients exhibited a marked decrease in IL-8 release in response to LPS and TNF-α stimulation. Interestingly, neutrophils isolated from CTx patients yielded an 88% decrease in their capacity to release IL-8 under LPS stimulation (powerful proinflammatory mediator), whereas this reduction was less significant in response to weaker proinflammatory mediators (fMLP and TNF-α (Figure 2). There were no significant differences in any of the study parameters in patients with CAV (Table 2).

## Discussion

In this clinical investigation, we report a decrease in circulating levels of VEGF-A_165_ but no significant changes in plasma levels of the proinflammatory cytokine IL-8 and the anti-inflammatory cytokine IL-1RA in stable long-term CTx recipients. Isolated neutrophils from CTx patients yielded a marked attenuation in the release of VEGF-A_165_, IL-1RA and IL-8 in response to most agonists. Although basal and stimulated levels of VEGF-A_165_ were consistently lower in patients with CAV, this difference did not reach statistical significance.

VEGF superfamily members play a pivotal role on angiogenesis and inflammation helping to maintain healthy adult vascular function and homeostasis [32,33]. However, VEGF analogs (predominantly VEGF-A_165_) also possess significant proinflammatory properties, by their capacity to increase vascular permeability and to promote the adhesion and transvascular migration of leukocytes [34,35]. There has been little data on the characterisation and on the role of VEGF analogs following CTx. Earlier work from Torry et al. showed that cardiac immunreactivity against VEGF-A isoforms increased significantly (predominantly VEGF-A_165_ isoform) in cardiac allograft concomitantly with fibrin depositions as well as the presence of macrophages and neutrophils [17]. Recently, Gramley and coworkers reported a significant increase in cardiac fibrosis over time in specimens of endomyocardial biopsies [16]. Using an immunohistochemistry approach (selective antibody for VEGF-A isoforms), this group reported a parallel increase in cardiac VEGF-A in these biopsy specimens. This investigation suggested that myocardial hypoxia occurs in long-term CTx recipients and that VEGF-A may provide an adaptive mechanism to reduce hypoxic stress following CTx.

Herein we report a decrease in circulating VEGF-A_165_ and a marked attenuation in the capacity of neutrophils to release VEGF-A_165_. This contrasts with the finding from Daly et al. [18] who reported an increase in circulating levels of VEGF-C and VEGF-A_165_ in patients with CAV. The reasons for these discrepancies with our data remain speculative. Nevertheless, in the latter study the data from cardiac transplant recipients were not compared with those from healthy control subjects and about 80% of patients were treated with cyclosporine-based immunoprophylaxis. In addition about 1/3 of patients received mycophenolate acid-based treatments. However, one might speculate that an increase in cardiac (cardiomyocytes) VEGF-A isoforms may be associated with a decrease in circulating level because of the avidity of the injured myocardium to VEGF. The decreased release of VEGF-A_165_ by stimulated neutrophils may also be related to some “exhaustion” of the neutrophils related to an increased demand and/or the inhibition of corresponding synthesis mediated by the immunosuppressors [36-38]. Interestingly, circulating levels of IL-8 were not significantly decreased in patients suggesting that chronic immunosuppression may not solely explain these observations. Our data would be in agreement with Spisani et al. reporting no significant impacts of cyclosporine A on either basal or agonist-stimulated neutrophils intracellular calcium concentrations [36]. The mechanisms for these observations and the physiological impacts of a decrease in VEGF-A_165_ in these high-risk patients deserve further investigations.

In this study we observed no significant decrease in plasma levels of IL-1RA and IL-8 in CTx patients compared with the HC subjects. In contrast, the capacity of stimulated neutrophils to increase the release of these cytokines was significantly attenuated following CTx. Various cytokines including IL-1, IL-6 and IL-8 play a significant role on vascular injury and inflammation [22,39]. IL-1 and TNF-α are known to induce the release of IL-8 [22], the latter promoting the migration of neutrophils to the inflammatory site. IL-8 played a significant role on ocular inflammation and angiogenesis in conjunctiva [30] and on atherogenesis [22,40] and its inhibition using a specific [22] antibody reduced ischemia reperfusion injuries in the heart [41]. IL-1RA belongs to the IL-1 family and binds to IL-1 receptors, thereby antagonising the inflammatory effects of IL-1α and –ß [42]. Immune cells such as neutrophils, can secrete IL-1RA [43,44] and the latter may prevent the proinflammatory effects of IL-1 [42]. The balance between IL-1 and IL-1RA systemically or locally plays an important role in many diseases such as arthritis, renal failure, and cancer [42,45-47]. In early post renal transplant patients, reduced IL-1RA is associated with delayed graft function [48] and IL-1RA gene transfer inhibits graft rejection in an experimental model of corneal transplantation [49]. In addition, low post-transplantation IL-1RA levels correlate with engraftment syndrome after autologous stem cells transplantation in plasma cell neoplasms [50]. Basal and stimulated IL-1RA levels had not been investigated following CTx.

Recently, published work from our group reported a plasmatic increase of various cytokines including IL-1 and IL-6 within the first 12 weeks following de novo cardiac transplantation [1]. Plasma levels for these specific cytokines decreased significantly but did not reach levels observed in healthy control subjects at 12 months. We also reported some elevation of plasminogen activator inhibitor-1 (PAI-1), fibrinogen, and high sensitivity C-reactive protein (hsCRP) in long-term CTx recipients [2]. Levels for these specific markers were only mildly elevated and cytokines were not measured in these patients. Unfortunately VEGF was not measured in these studies. In the present investigation, we expanded these latter observations in CTx recipients by reporting minimal changes in basal levels of two potentially physiologically relevant cytokines. In contrast, we observed a profound decrease in the release of VEGF-A_165_ along with these two cytokines by stimulated neutrophils. The mechanisms inducing the decrease in both IL-8 and IL-1RA remain unknown. However, we may speculate that a chronic state of inflammation may contribute to decrease the potential release of inflammatory markers by the neutrophils. Similar behaviour has been reported with other cytokines such as TNF-α in heart failure [51]. The attenuations in IL-1RA release may also suggest that these patients may fail to compensate for an elevation in many proinflammatory cytokines. To what extent these findings are related to chronic immunosuppression or other abnormalities in cytokine regulation is a matter for future investigations.

### Study limitations

This clinical investigation reported novel and significant findings on VEGF-A_165_ and on selected cytokines in CTx recipients. Only VEGF-A_165_ was measured. Also the investigations of all VEGF subsets such as VEGF-A and -C would have provided more complete data and insights in these patients. It is to mention that the neutrophils do not have the capacity to release IL-1ß following a proinflammatory stimulation (including LPS treatment; 0–24 hours stimulation period); even though there is an increase in IL-1ß synthesis [27]. In addition, the detection of IL-1ß in the serum and plasma of healthy volunteers, using a high-sensitivity IL-1ß ELISA kit is either non-detectable or minimally detectable in only 9 to 25% of the measured samples. Thus, we did not measure IL-1ß and as such the ratio of IL-1ß/IL-1RA could not be reported and discussed. More extensive work on other cells playing a significant role on vascular biology such as the monocyte and lymphocyte will be justified in future investigations. In contrast to healthy controls, cardiac transplant patients exhibited a high prevalence of hypertension, dyslipidemia and some degree of renal failure. In addition all transplant recipients were on various immunosuppressive regimen and most of them were on antihypertensive drugs and on statins. It is likely that these medical conditions and drug used may have played a role on neutrophil responses. Nevertheless this potential bias could not be avoided in this clinical investigation. Also despite adequate age-matching our study population were not sex-matched. The impact of gender on these parameters would be a matter for future works. In this study, patients with CAV exhibited lower but non-significant values for all markers in response to simulation. However, from all 18 patients, only a small group of our study population exhibited some mild degree of CAV. As such our report does not allow us to conclude about the impact of CAV on these findings.

In conclusion, CTx recipients exhibit a marked reduction in circulating VEGF-A_165_ as well as in neutrophil mediated release in VEGF-A_165_. The mechanisms and physiologic impacts of these findings and their relationship with various severity of CAV deserve additional investigations.
